# Anal squamous cell carcinoma: Impact of radiochemotherapy evolution over years and an explorative analysis of MRI prediction of tumor response in a mono-institutional series of 131 patients

**DOI:** 10.3389/fonc.2022.973223

**Published:** 2022-10-24

**Authors:** Marco Lorenzo Bonù, Salvatore La Mattina, Navdeep Singh, Cristian Toraci, Luigi Spiazzi, Fabrizia Terraneo, Fernando Barbera, Paola Vitali, Francesco Frassine, Andrea Guerini, Luca Triggiani, Davide Tomasini, Vittorio Morelli, Jessica Imbrescia, Jacopo Andreuccetti, Barbara Frittoli, Frida Pittiani, Luigi Grazioli, Nazario Portolani, Luca Nicosia, Domenico Albano, Francesco Bertagna, Stefano Maria Magrini, Michela Buglione

**Affiliations:** ^1^ Department of Radiation Oncology, Istituto del Radio O. Alberti, University of Brescia and Spedali Civili Hospital, Brescia, Italy; ^2^ Department of Medical Physics, Spedali Civili Hospital, Brescia, Italy; ^3^ Department of General Surgery, Spedali Civili Hospital, Brescia, Italy; ^4^ Department of Radiology, Spedali Civili Hospital, Brescia, Italy; ^5^ Department of General Surgery, University of Brescia and Spedali Civili Hospital, Brescia, Italy; ^6^ Advanced Radiation Oncology Department, Cancer Care Centre, IRCCS Sacro Cuore Don Calabria Hospital, Negrar, Italy; ^7^ Department of Nuclear Medicine, ASST Spedali Civili di Brescia and Brescia University, Brescia, Italy

**Keywords:** anal cancer (AC), radiotherapy–chemotherapy, IMRT (intensity modulated radiation therapy), predictive modeling, mri

## Abstract

**Introduction:**

Radiochemotherapy (RCHT) for the treatment of anal squamous cell carcinoma (ASCC) has evolved dramatically, also thanks to intensity-modulated RT (IMRT) and 3D image guidance (3D IGRT). Despite most patients presenting fair outcomes, unmet needs still exist. Predictors of poor tumor response are lacking; acute toxicity remains challenging; and local relapse remains the main pattern of failure.

**Patients and methods:**

Between 2010 and 2020, ASCC stages I–III treated with 3D conformal radiotherapy or IMRT and CDDP-5FU or Mytomicine-5FU CHT were identified. Image guidance accepted included 2D IGRT or 3D IGRT. The study endpoints included freedom from locoregional recurrence (FFLR), colostomy free survival (CFS), freedom from distant metastasis (FFDM), overall survival (OS), and acute and late toxicity as measured by common terminology criteria for adverse events (CTCAE) version 5.0. An exploratory analysis was performed to identify possible radiomic predictors of tumor response. Feature extraction and data analysis were performed in Python™, while other statistics were performed using SPSS^®^ v.26.0 software (IBM^®^).

**Results:**

A total of 131 patients were identified. After a median FU of 52 months, 83 patients (63.4%) were alive. A total of 35 patients (26.7%) experienced locoregional failure, while 31 patients (23.7%) relapsed with distant metastasis. Five year FFLR, CFS, DMFS and PS resulted 72.3%, 80.1%, 74.5% and 64.6%. In multivariate analysis, 2D IGRT was associated with poorer FFLR, OS, and CFS (HR 4.5, 4.1, and 5.6, respectively); 3DcRT was associated with poorer OS and CFS (HR 3.1 and 6.6, respectively). IMRT reduced severe acute gastro-intestinal (GI) and severe skin acute toxicity in comparison with 3DcRT. In the exploratory analysis, the risk of relapse depended on a combination of three parameters: Total Energy, Gray Level Size Zone Matrix’s Large Area High Gray Level Emphasis (GLSZM’s LAHGLE), and GTV volume.

**Conclusions:**

Advances in radiotherapy have independently improved the prognosis of ASCC patients over years while decreasing acute GI and skin toxicity. IMRT and daily 3D image guidance may be considered standard of care in the management of ASCC. A combination of three pre-treatment MRI parameters such as low signal intensity (SI), high GLSZM’s LAHGLE, and GTV volume could be integrated in risk stratification to identify candidates for RT dose-escalation to be enrolled in clinical trials.

## Introduction

Anal squamous cell carcinoma (ASCC) is a rare disease with an increasing incidence. HPV infection is associated with up to 93% of cases (1–4). Immuno-evasion plays a significant role in cancer transformation and gives an explanation for a higher incidence of cancer in HIV patients (3). Sphincter-preserving therapy represented by radiochemotherapy (RCHT) is the standard of care for stage I–III disease (5, 6). Clinical outcomes are dependent on stage. Overall, clinical trial best arms showed 5-year overall survival (OS) of approximately 78% and 5-year disease-free survival (DFS) of between 67% and 74% (4, 6, 7). Functional outcomes are crucial in ASCC, with colostomy-free survival (CFS) achieved at 5 years in approximately 70% of patients. Patterns of relapse are mainly local, with a risk of local failure of about 20%–24% at 5 years. Abdominoperineal resection (APR) represents the salvage option, is burdened by high morbidity, and results in further local failure in 40%–50% of cases (8–11). Patients with systemic relapse or persistent disease are candidates for systemic therapy, with an expected response rate of about 59% for the most active regimens. Unfortunately, the duration of response is suboptimal, with a median OS of 20 months (12). Thus, as local failure remains the main site of relapse, strategies to optimize local control and functional outcomes are needed. Intensity-modulated radiotherapy (IMRT) and simultaneous integrated boost (SIB) planning seem to reduce gastro-intestinal (GI) treatment morbidity (7). Magnetic resonance imaging (MRI) is recommended as the imaging modality of choice for loco-regional staging of ASCC and plays a key role in radiation therapy planning. The optimal radiotherapy dose and fractionation remain an open issue, with no clear advantage to more aggressive schedules (5). Moreover, predictive tools for response to RCHT are lacking in ASCC.

Recently, MRI features have been exploited for treatment response prediction in different oncological settings. However, given the rarity of the disease, there is a lack of data concerning the MRI signature as a predictive tool in ASCC (13).

Our study aims to report the clinical outcomes of ASCC patients treated in a high-volume center. Our series will focus on the evolution of RCHT treatment techniques and schedules, with a particular interest in their impact on efficacy and functional outcomes. Moreover, pre-treatment diagnostic MRI will be exploited to identify a surrogate of biological behavior predicting tumor radiosensibility and local relapse.

## Material and methods

The present is a mono-institutional, retrospective analysis. Informed consent was obtained for each patient prior to inclusion in this study. The study inclusion criteria were as follows:

− Histologic diagnosis of ASCC in clinical stages I-III, locally staged with pelvic MRI and/or endorectal ultrasound.

− For systemic staging purposes, whole body contrast enhanced CT scan was used. PET TC was permitted but not mandatory.

− All patients were screened for chemotherapy with a complete cardiological assessment consisting of electrocardiography, echocardiography, and a medical visit. Furthermore, all patients were tested for DPYD gene mutation prior to chemotherapy administration.

− ECOG (Eastern Cooperative Oncology Group criteria) Performance status ≤2.

### Radiotherapy (RT) planning

All patients should have been simulated in a prone or supine position, with a Combifix (CIVCO^®^) or Belly board (CIVCO^®^). A 3D free-breathing CT scan with 2–3 mm slices was acquired. Imaging co-registration with diagnostic MRI or PET CT was permitted. When indicated, a contrast-enhanced CT scan was performed. In the case of perianal skin involvement, a radiopaque marker was placed, and a secondary CT scan was acquired to help discriminate the caudal limit of Gross Tumor Volume (GTV). All patients were simulated as per protocol with a comfortably filled urinary bladder. GTV_T and GTV_N were defined using all clinical and radiological data as well as rigid imaging coregistration when available. Clinical Target Volume (CTV) was defined according to RTOG consensus panel contouring guidelines for 3DCRT and AGITG guidelines for IMRT (14, 15).

Image-guided radiotherapy (IGRT) was mandatory for all patients before each fraction. Patients treated before 2015 were verified with portal imaging. Subsequently all patients have been verified with 3D IGRT (cone beam CT or Megavoltage CT).

### Follow-up protocol and assessment of tumor response

Physical examination, complete blood count, liver function test, total bilirubin, INR, ALP, GGT, and creatinine were performed 28–30 days after RT and then at each follow-up. Patients were followed every 3 months for the first year after treatment, every 4 months for the second year and every 6 months thereafter. Assessment of tumor response followed RECIST criteria and was mandatory with MRI or endorectal ultrasound at 3 and 6 months, 9 and 12 months. Following that, patients underwent a clinical examination as well as a contrast-enhanced whole-body CT scan. Lower abdomen contrast enhanced MRI was mandatory in the case of clinical or radiological suspicion of locoregional relapse; endoanal ultrasound or 18-fuorodeoxyglucose-positron emission tomography (FDG-PET/CT) was requested at the choice of the investigator. An anoscopy with biopsy was performed in the case of a positive MRI but never before 6 months after the end of radiotherapy (5).

### Study endpoints and statistical analysis

The primary endpoint of the study was freedom from local recurrence (FFLR), defined as the time from RT to local progression inside the Planning Target Volume (PTV) or last follow-up, diagnosed radiologically and confirmed Cito/histologically. Importantly, clinical and radiological responses were scored at 3 and 6 months, given the evidence of best tumor response at 6 months described in the literature (5).

− Colostomy free survival (CFS), defined as the time from RT to colostomy or last follow-up, colostomies after treatment were counted, including pre-treatment colostomies not reversed within 8 months after starting treatment.

− Freedom for salvage therapy (FFS), defined as the time from RT to salvage surgery (APR, other local resection, or salvage lymph node dissection), or last follow-up.

− Freedom from distant metastasis (FFDM), defined as the time from RT to systemic disease progression diagnosed radiologically or cito/histologically or last follow-up.

− PFS is defined as the time from RT to disease progression (any site) diagnosed radiologically or Cito/histologically or last follow-up.

− OS is defined as the time from RT to death for any cause or last follow-up.

− Acute and late toxic effects were assessed with the National Cancer Institute Common Terminology Criteria for Adverse Events (CTCAE), version 5.0; acute toxicity was defined as any adverse event occurring from the beginning of the RT up to 90 days afterwards. Late toxicity was defined as any adverse event occurring at least 91 days after RT.

The distribution of the different clinical and therapeutic features was compared with the chi square test.

Univariate analysis was performed to identify variables with a statistically significant impact on each outcome. Survival analysis was performed with the Kaplan–Meier method, and the log rank test was applied to compare the effect of the individual variables on the different outcomes.

The variables analyzed in the univariate model were as follows: sex, age, histologic tumor characteristics, tumor grade, HPV status, anatomical subsite (anal canal, perianal skin), presence of fistula, T stage, N stage, stage group, tumor ulceration, tumor diameter, HIV infection, smoke, chemotherapy (CHT) scheme, chemotherapy compliance (defined as the administration of at least 75% of the ideal dose), radiotherapy technique, radiotherapy schedule consisting of sequential boost versus simultaneous integrated boost (SIB), type of image guidance (2D versus 3D), and radiotherapy dose (biologic effective dose calculated using an alpha/beta ratio of 10 for the tumor). Moreover, an analysis of the impact of unplanned treatment interruption (defined as interruption of RT for 5 or more days) was performed. For this study, all patients were retrospectively re-classified following the 8th Edition of Tumor-Node-Metastasis Classification. A Cox proportional hazards model was planned to find independent predictors of local recurrence and survival. Univariate analysis led to the selection of variables to consider as predictors. All tests were two-tailed, and a probability value of less than.05 was considered statistically significant. The collected data were analyzed using IBM^®^ SPSS^®^ v.26.0 software.

− An exploratory analysis was performed to identify possible radiomic predictors of tumor response to RCHT. All available pre-treatment MRIs were collected.

GTV was contoured on T2-weighted MR. For quantitative image analysis, after contouring, every image sequence was resampled to an isotropic voxel of 2 mm, using trilinear interpolation. No smoothing filter was applied. Radiomic features of GTV were extracted from the resampled image sequences according to the IBSI initiative (16), with the goal of fitting radiomic features to loco-regional recurrence. The IBSI defines a set of 169 features. which are too many for two reasons. First, our data set contains just thirty-one cases, and the fitting of 31 samples over more than a hundred independent variables is an ill-posed problem under any circumstances. Second, we expect that just a small subset of features—or their combination—is related to loco-regional recurrence. For these reasons, we applied a Principal Components Analysis (PCA) (17), which creates a new set of independent variables (*components*) with a reduced number of variables but minimal loss of information. PCA works by first rescaling all the input variables such that they have zero mean and unit variance. This reduces the bias caused by variables with high scale differences. Then the algorithm searches for a direction in the variable space such that the variance is maximized. This direction is a linear combination of the original variables. This direction is called the first *principal component*, and the amount of total variance along this variance is the variance *explained* by this principal component. The second principal component is built in the same way, but this time the algorithm searches for a direction that is orthogonal to the previous and explains just the variance that is not already explained. The process repeats until all the variance is explained and the number of components matches the number of initial variables. Since we aim at classifying groups different of data we want to look where the data are inhomogeneous, (i.e., where the variance is higher) therefore in PCA analysis just the first components are considered. PCA components were fitted to classify loco-regional recurrence using a Support Vector Machine (SVM) with a linear kernel (18). A SVM is the simplest machine learning classifier and was chosen because it is the most reliable when applied to a small data set. A k-fold cross validation with five folds was used to fine tune two parameters for our classification: the number of PCA components and the balance between fitting accuracy and noise tolerance in SVM. The area under the receiver operator characteristic curve (AUC) was used to assess the best fitting result. No validation was performed on any of the fittings due to the lack of enough cases. Feature extraction and data analysis were performed in Python ™ using well-established machine learning and imaging libraries (19–21).

## Results

### Patients’ characteristic

From January 2010 to December 2020, 131 patients treated with RT for ASCC and meeting the inclusion criteria were identified. Median age was 64 years (a range of 39–86); most patients (70.2%) were female and presented a diagnosis of an HPV-positive (76%), true anal canal squamous cell carcinoma (79,4%). Nineteen patients (14.5%) were HIV positive and on HAART, and 52.3% were active or former smokers.

Concerning tumor stage, 58% of patients presented in stage III, 29.8% in stage II and 12.2% in stage I. Median primary cancer diameter was 4 cm. In eight cases (6.1%) there was evidence of fistulization between primary tumor and perianal skin. A pre-treatment colostomy was performed in 25 patients (19.1%) because of tumor bulk and the risk of occlusion.

A total of 81 patients (61.9%) were staged with pelvic MRI, the rest with endorectal ultrasound. For systemic staging, the whole series was staged with a contrast enhanced whole body CT scan. In 22 cases (16.8%), an additional fluorodeoxyglucose PET CT scan was performed.

### Treatment characteristics

A total of 125 patients were simulated in the supine position with a combifix immobilization support, while six patients were simulated in the prone position with a bellyboard. Fifty-nine patients (45%) were treated with 3D conformal radiotherapy technique (3DCRT), while 72 patients (55%) were treated with intensity modulated radiotherapy (IMRT), including step and shoot IMRT, helical IMRT, and volumetric modulated arc therapy (V-MAT). IGRT consisted of 2D portal imaging for 62 patients (47.3%) and 3DIGRT with Cone Beam CT (CBCT) or Megavoltage CT (MVCT) for the rest of the patients treated with IMRT.

Radiotherapy planning and target definition followed RTOG 9811, with a schedule consisting of 45 Gy on elective volumes followed by a sequential boost to locoregional disease up to 59.4 Gy in 33 fractions for 122 (93.1%) and RTOG 0529, with a schedule consisting of 46.2 Gy on elective volumes and up to 56 Gy with SIB in nine patients (6.9%). For both schedules, biologic effective dose (BED) to the primary tumor and positive lymph nodes ranged between 67.2 Gy and 70.01 Gy, considering an alpha–beta ratio of 10 for the tumor.

For 25 patients (19.1%) treated with 3DCRT between 2010 and 2011, a split-course schedule with a 15-day gap before the RT boost was performed. In seven cases, an unplanned protocol violation with treatment interruption for more than 5 days occurred.

Chemotherapy was administered to 113 patients (86.3%) and consisted of 5FU-CDDP for 52 patients (39.7%) and Mitomycin-5FU for 61 patients (46.6%). The reasons for chemotherapy omission in 18 patients were age >85 years, creatinine clearance <30 ml/min, or severe active cardiopathy. Compliance with the chemotherapy schedule was achieved in 100 patients (88%).

Whole-patient and treatment characteristics are presented in **Table 1**.

**Table 1:** Whole patient and treatment characteristics.

Variable	N (%)
AGE	
<65	69 (52.7%)
≥65	62 (47.3%)
Sex	
M	39 (29.8%)
F	92 (70.2%)
Histological subtype	
Squamous cell carcinoma	104 (79.4%)
Squamous cell basaloid carcinoma	27 (20.6%)
Smoking History	
No	56 (42.7%)
Former or active	75 (37.3%)
HPV state	
negative	31 (24%)
positive	100 (76%)
HIV	
Negative	112 (85.5%)
Positive	19 (14.5%)
Tumor stage	
I	16 (12.2%)
II	39 (29.8%)
III	76 (58%)
Fistula	
No	123 (93.9%)
Yes	8 (6.1%)
Pre-treatment colostomy	
No	106 (80.9%)
Yes	25 (19.1%)
Local staging	
Pelvic MRI	81 (61.9%)
Endorectal ultrasound	50 (38.1%)
PET CT	
no	109 (83.5%)
yes	22 (16.5%)
Treatment set-up	
supine	125 (95.4%)
prone	6 (4.6%)
RT technique	
3DCRT	59 (45%)
IMRT (step and shoot, V-MAT, Helical)	72 (55%)
IGRT	
Portal Imaging (2D IGRT)	62 (47.3%)
Cone Beam CT (3D IGRT)	69 (52.7%)
RT Schedule	
Sequential	122 (93.1%)
Simultaneous integrated boost	9 (6.9%)
Split Course	
No	106 (80.9%)
yes	25 (19.1%)
Unplanned RT interruption	
No	124 (94.7%)
yes	7 (5.3%)
Chemotherapy schedule	
No cht	18 (14.2%)
Mitomicine-5FU	61 (46.1%)
CDDP-5FU	52 (39.7%)
Compliance with CHT	
no	13 (12%)
yes	100 (88%)

3DCRT, 3D conformal radiotherapy; IMRT, intensity modulated radiotherapy; V-MAT, volumetric-modulated ARC therapy; IGRT, image-guided radiotherapy.

### Clinical outcomes

After a median FU of 52 months, 83 patients (63.4%) were alive. A total of 35 patients (26.7%) experienced locoregional failure, while 31 patients (23.7%) relapsed with distant metastasis.

**Figure 1:**
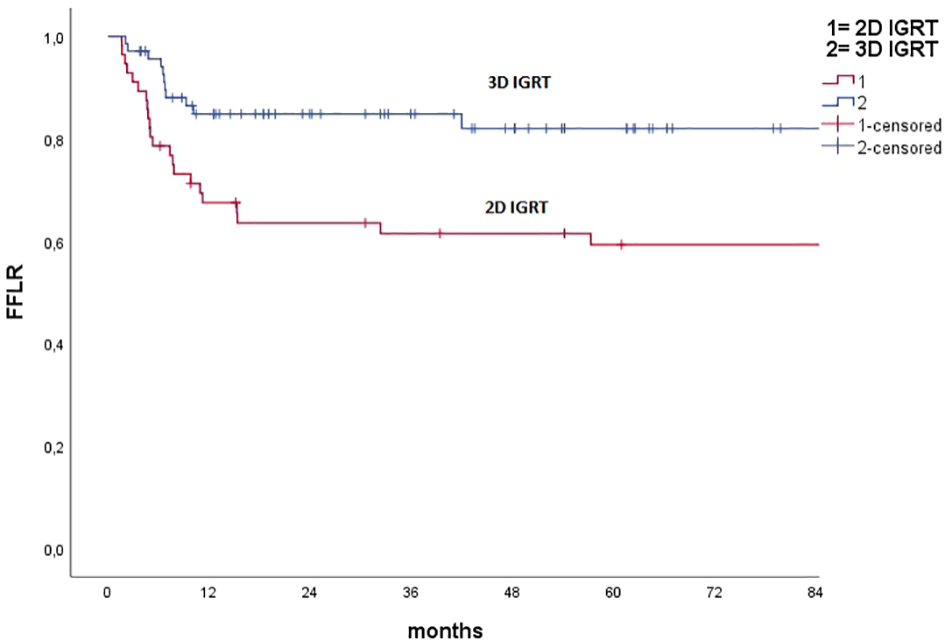
Kaplan–Meier curve concerning freedom from local recurrence for pts treated with 2D IGRT (portal imaging) and 3D IGRT.

**Figure 2:**
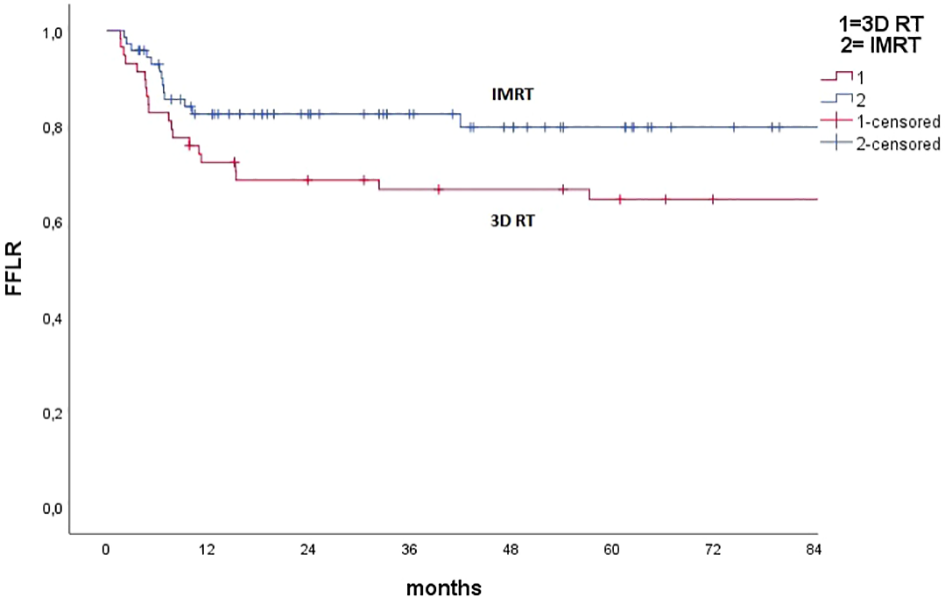
Kaplan–Meier curve concerning freedom from local recurrence for pts treated with 3D conformal Radiotherapy (3D RT) versus IMRT (all techniques).

Median FFLR was not reached; 3 and 5 year FFLR results were 74.3% and 72.3%, respectively. At univariate analysis, factors predicting locoregional relapse were: male sex (*p <*0.001), true squamous cell carcinoma versus basaloid ASCC (*p* = 0.01), tumor fistulization (*p* = 0.012), N+ nodal stage (p = 0.017), high stage group (*p* = 0.042), HIV positivity (*p* = 0.022), CHT schedule with CDDP-5FU (*p* = 0.04), split course schedule (*p* = 0.03), unplanned treatment interruption (*p* =0.002), 2D versus 3D IGRT (*p* =0.008). Furthermore, 3D technique versus IMRT showed a trend toward worse local control for 3D (*p* =0.065). **Figures 1**, **2** report Kaplan–Maier curves concerning FFLR for patients treated with 3D versus IMRT technique and with 2D versus 3D IGRT. Multivariate analysis confirmed male sex, true squamous cell carcinoma, presence of fistula, high stage group, unplanned RT interruption, and portal imaging as factors associated with poorer local control.

Concerning CFS, at last follow-up, 22 patients (16.5%) required a colostomy due to tumor relapse, while 23 patients (17.3%) did not close the colostomy placed before treatment. The median CFS was not reached with a 3 and 5 year CFS of 80.1%. At univariate analysis, factors negatively affecting CFS were male sex (*p <*0.001), true squamous cell carcinoma (*p* = 0.004), tumor fistulization (*p* = 0.012), N+ disease (p = 0.029), HIV positivity (*p* = 0.005), poor compliance with CHT schedule (*p* = 0.031), split course schedule (*p* = 0.049), and 2D IGRT (*p* =0.0013). Multivariate analysis confirmed male sex, true squamous cell carcinoma, presence of fistula, poor compliance with cht schedule, unplanned RT interruption, and 2D IGRT as factors associated with poorer colostomy free survival.

The median FFDM was not reached, with a 3 and 5-year FFDM of 76% and 74.5%. Univariate analysis showed that male sex (p = 0.002), N+ (p <0,001), high stage group (p <0.001) were associated with worse FFDM. Multivariate analysis confirmed male sex and high stage group as independent prognostic factors.

The median OS resulted in 99 months, with a 3- and 5-year OS of 79.2% and 64.6%, respectively. At univariate analysis, variables associated with a poorer OS were poor compliance to the CHT schedule (p = 0.019), 3D versus IMRT technique (p <0.001), split course schedule (p <0.001), 2D versus 3D IGRT (p <0,001). Multivariate analysis showed 3D technique and 2D IGRT as independent factors associated with worse OS.

The median FFS was not reached, with a 3- and 5-year FFS of 80% and 78%, respectively. Of the 35 patients that experienced a locoregional failure, 26 were eligible for salvage therapy. In 23 patients, an APR was performed, in three cases, a salvage inguinal lymphadenectomy. In 14 of 26 cases (54%), salvage was successfully accomplished with patients free from local recurrence at last follow-up. The median freedom from loco-regional progression after surgical salvage was 19 months. Multivariate analysis results for all clinical outcomes are summarized in **Table 2**.

**Table 2:** Multivariate analysis results concerning FFLR, CFS, FFS, FFDM, and OS.

Variable	FFLR			CFS			FFS			FFDM			OS		
	Adj O.R.	95% C.I.	*p*	Adj O.R.	95% C.I.	*p*	Adj O.R.	95% C.I.	*p*	Adj O.R.	95% C.I.	*p*	Adj O.R.	95% C.I.	*p*
Male sex	3.5	1.7–7.1	<0.001	5.5	2–15.1	0.001	2.1	1.01–4.5	0,045	2.4	1.2–4.9	0,015	NS		
True squamous cell carcinoma	1.4	1.1–2.0	0.036	1.36	1.1–2.2	0.033	NS			NS			NS		
Tumor fistulization	4.5	1.5–13.4	0.006	9.8	2–48.4	0.005	NS			NS			NS		
N+	NS			NS			NS			4.9	2–12.2	0,001	NS		
High stage group	2.56	1.3–4.9	0.006	NS						NS			NS		
HIV +	NS			NS			NS			NS			NS		
CDDP-5FU cht	NS			NS			NS			NS			NS		
Split course schedule	NS			NS			NS			NS			NS		
Cht compliance	NS			5.3	1.8–17	0.003	NS			NS			NS		
Unplanned treatment interruption	6.2	2.1–17.9	0.001	80	6.7–950		NS			NS			NS		
2D vs 3D IGRT	4.5	1.9–10.4	0.001	4.1	1.1–14.9	0.031	NS			NS			5.6	1.7–18.1	0.01–2
3DCRT vs IMRT	NS			3.1	0.96–9.9	0.059	NS			NS			6.6	1.5–29.1	0.004

3DCRT, 3D conformal radiotherapy; IMRT, intensity modulated radiotherapy; V-MAT, volumetric-modulated ARC therapy; IGRT, image-guided radiotherapy; N+, positive nodes; HIV+, HIV positive; NS, not statistically signficant.

### Time-tumor response

Of the 94 patients free from locoregional recurrence, 37 patients were classified with MRI or endorectal ultrasound as partial responders at 3 months after RT, being classified as complete responders at 6 months. Of the 35 patients that experienced a loco-regional failure, only two patients were judged to have a clinical and radiological complete response at 3 and 6 months. Clinical examination showed a concordance with local imaging results at three and six months in 70% and 89% of cases, respectively.

### Toxicity

One case of acute G4 hematologic toxicity occurred (neutrophil count decreased). Concerning acute G3 toxicities, 64 patients experienced G3 radiation dermatitis, 38 patients experienced G3 anal pain, and 10 patients showed a neutrophil count decreased. Rare G3 toxicities included anal haemorrhage, urinary retention requiring foley catheter placement until the end of treatment, vomiting, and noninfective cystitis.

Concerning late toxicity, only one case of G3 toxicity occurred, consisting of a case of anal hemorrhage without disease recurrence requiring endoscopic coagulation. Three patients experienced G2 fecal incontinence. However, in such patients, continence was impaired before RT. Other rare G2 toxicities were represented by anal pain, dyspareunia, radiation fibrosis of the perianal region, hemorrhage, and increased urinary frequency.

An analysis of the impact of the 3D technique versus IMRT on acute and late toxicity was performed.

In the chi-square test, IMRT reduced G3 or higher GI acute toxicity from 41.4% to 23.9% (p = 0.015) and G3 or higher skin toxicity from 59.3% to 41.7% (p = 0.033). No impact on acute GU and late GI and GU toxicity was found.

Whole acute and late maximum toxicity are presented in **Table 3**.

**Table 3:** Maximum acute and late toxicity.

TOXICITY TYPE	ACUTE			LATE			
	G1–2	G3	G4	G1	G2	G3	G4
GI							
anal pain	56	38	NA	10	2	0	NA
diarrhea	8	0	0	18	0	0	0
hemorrhage	2	1	0	6	2	1	0
vomiting	0	1	0	0	0	0	0
Fecal incontinence	0	0	NA	15	3	0	NA
GU							
Dysuria	28	NA	NA	4	NA	NA	NA
Noninfective cystitis	1	1	0	1	1	0	0
Urinary incontinence	2	0	NA	4	0	0	NA
Urinary frequency	4	0	NA	1	1	0	NA
Urinary retention	0	1	0	0	0	0	0
Dyspareunia	NA	NA	NA	10	2	0	NA
SKIN							
Radiation dermatitis	60	64	0	0	0	0	0
Radiation fibrosis	NA	NA	NA	20	6	0	0
OTHER							
Anemia	12	0	0	0	0	0	0
Neutrophil count decreased	20	10	1	0	0	0	0

NA, Not Appliable.

### Exploratory radiomic analysis

Thirty-one patients presented an available diagnostic MRI for exploratory analysis purposes. Of this subgroup, the majority of patients presented in stage III; all patients were treated in supine position with IMRT and daily 3D IGRT to a total dose of 59.4 Gy on gross disease and elective dose of 45 Gy with sequential schedule concurrently with Mitomycin-5FU chemotherapy.

**Figure 3:**
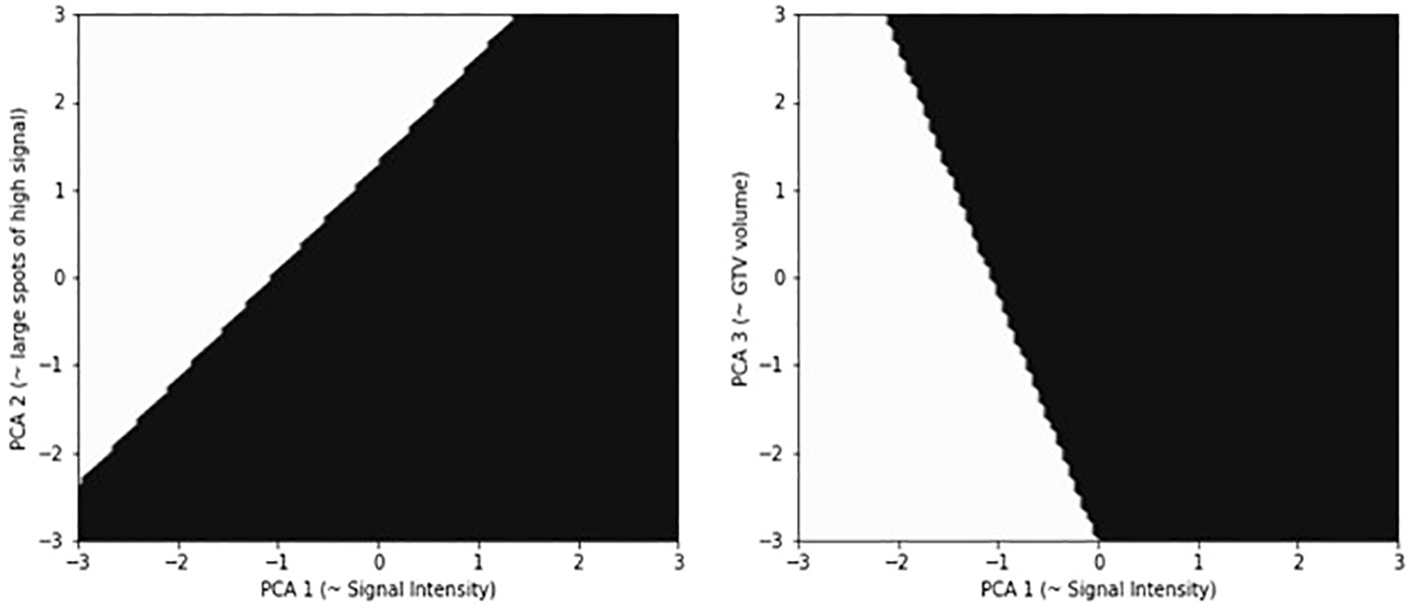
Results of the 3-component SVM classification. PCA component are normalized such that data have zero mean and variance one. In the image on the left, classification is based on Signal Intensity versus LAHGLE, in the image on the right is based on Signal intensity versus GTV. The white area is where the model predicts a relapse, the black area is where it predicts no relapse. For simplicity of explanation, we shall assume that PCA1 is SI, PCA2 is GLSZM’s LAHGLE, PCA3 is GTV volume. the relapses are on the left side of both panes. Since both panes have SI as horizontal axis, it is confirmed that a lower SI correlate with a higher risk of relapse. In the left pane the vertical axis is LAHGLE, and relapses are in the upper part, higher LAHGLE has a positive correlation with relapse. In the right pane GTV is on the vertical axis and with a similar reasoning we can conclude that a smaller GTV volume correlates with relapse. The risk of relapse depends on a combination of the three parameters since the cut-off between relapse and non-relapse is never a horizontal or vertical line.

Eight patients presented with a locoregional disease relapse, and 23 patients were free from locoregional disease at last FU. Mean GTV volume resulted in 30 cc in patients with relapse and 33 cc in patients free from disease, respectively (*p* = ns). PCA applied to T_2_ images shows that the first component (PCA1) is enough to explain more than 99% of the variance, and total energy is the main contributor to this component (98%). Total energy is a measure of MRI signal intensity (SI). From this first level analysis, we may assume that tumors with a lower SI correlate with a higher risk of recurrence. A rule of thumb for PCA analysis is to keep enough components to explain 95% of the variance, which helps minimize noise contribution. With this logic, the first component of our PCA should be the only one in our analysis. On the other hand, our SVM classifier had the best fitting with 2 to 3 components, the main contributor to PCA 2 being Gray Level Size Zone Matrix’s Large Area High Grey Level Emphasis (GLSZM’s LAHGLE) (99%), and GTV volume (97%) to PCA3. GLSZM’s LAHGLE is a second-order statistic of the MRI signal distribution, which may be interpreted as the number of relatively large areas of iper-intense signal inside the GTV. **Figure 3** shows our best classifier using three PCA components. The white area is where the model predicts a relapse; the black area is where it predicts no relapse. For simplicity of explanation, we shall assume that PCA1 is SI, PCA2 is GLSZM’s LAHGLE, and PCA3 is GTV volume. In **Figure 3**, the relapses are on the left side of both panes. Since both panes have SI as a horizontal axis, it is confirmed that a lower SI correlates with a higher risk of relapse. In the left pane, the vertical axis is LAHGLE, and relapses are in the upper part. Higher LAHGLE has a positive correlation with relapse. In the right pane, GTV is on the vertical axis and, with similar reasoning, we can conclude that a smaller GTV volume correlates with relapse. The risk of relapse depends on a combination of the three parameters since the cut-off between relapse and non-relapse is never a horizontal or vertical line.

## Discussion

Patterns of treatment for ASCC have evolved in the last decade thanks to the understanding of the detrimental impact of planned treatment interruptions such as split-course RT, optimization of chemotherapy schedule, and RT technique improvements. However, there are many unmet needs to be explored in ASCC.

Concerning clinical issues, 5Fu-Mitomycin represents the schedule of choice in ASCC. Our results support this assumption, with an advantage in terms of local control at univariate analysis for the 5FU-Mitomycin group over the CDDP-5FU group, although multivariate analysis did not confirm such a result. Interestingly, poor compliance with the chemotherapy schedule seems to impact more than the CHT scheme on OS and CFS. Moreover, also in our analysis, split-course RCHT is characterized by inferior outcomes. However, our results suggest that unplanned treatment interruptions also present a detrimental impact on local control, CFS, and OS. Adherence to RT and CHT could be challenging in such a context, given the impact of the treatment in terms of acute local toxicity. However, also given the detrimental effect shown by our results, this aspect deserves careful management, and every effort must be made to ensure adherence to the RT schedule and maintain CHT dose-intensity.

Management of HIV patients is an area of controversy, with some authors suggesting worse outcomes for this subgroup (22) and others suggesting similar outcomes in HIV-positive patients treated with HAART in terms of complete response and survival (23, 24). In our series HIV pts present a worse FFLR and CFS at univariate analysis, but HIV positivity seems not representing an independent factor associated with poorer FFLR and CFS at Multivariate analysis. Moreover, no impact of HIV has been found on OS.

High stage and the presence of nodal involvement are well known prognostic factors in ASCC. Concerning loco-regional disease burden, fistulisation is an under-reported condition, depending on the tumor patter of local spread or concurrent peritumoral inflammation. Little is known in literature about the management of such patients and their possible impact on tumor control. In our series, eight patients (6%) presented with anal-perianal tumor fistulization. Both univariate and multivariate analysis identified fistula to be related to a poorer FFLR and CFS. An explanation for this result could be found in the possible switch to a hypoxic microenvironment in the context of fistula, with a less effective cytotoxic effect of RCHT. Another hypothesis not to be excluded is that microscopic tumor spread in the context of tumor fistula could be less predictable and more complex to be described with MRI, and a more careful target volume definition could be required in such a context.

Concerning technical issues, IMRT has been demonstrated to present a fair acute and late toxicity profile (7). However, given the unique characteristics of IMRT, concern about marginal missing of microscopic disease spread exists, despite long terms results of a small clinical trial and retrospective series seems to show similar locoregional relapse events in comparison with 3DcRT data (25–27). Remarkably, comparison with 3DcRT has been made indirectly by RTOG 0529 with the best historical trial arms. In our series, IMRT represents an independent prognostic factor predicting an advantage in OS, while concerning FFLR and CFS, a trend toward an advantage has been found. Concerning IGRT, use of 3D IGRT such as Cone Beam CT is independently associated with better FFLR, CFS, and OS.

The role of IMRT and 3D IGRT could have been confounded by other factors. In fact, the development in the management of patients during recent years thanks to the understanding of the detrimental effect of split course schedule and the benefits of the introduction of Mitomycin-5FU could have impacted on clinical outcomes. In our view, thanks to MRI and PET CT based target volume delineation and the inter-fraction error reduction allowed by daily 3D IGRT, it has been possible to better manage pitfalls of IMRT such as marginal miss. Moreover, given the impact of locoregional failure on the prognosis of patients, locoregional effects reflected in advantage also in OS.

Also, our results support the observation of maximum tumor response at 6 months, showing a high concordance between clinical and radiological response at such a time interval.

Concerning toxicity, RCHT acute side effects are challenging, and also, in our experience, G3 perianal radiation dermatitis happens in approximately 50% of patients (7). Acute mild diarrhea happened in less than 10% of patients, also thanks to our set-up protocol requiring a comfortably full bladder set-up and, when necessary, the use of a belly board. As shown by other authors, also in our series, IMRT decreased acute ≥G3 GI and skin toxicity, contributing to optimizing therapeutic index.

Late toxicity was mild, with three cases of G2 fecal incontinence that happened in patients for whom this function was impaired by tumor burden before RCHT. No impact of IMRT on late toxicities was registered. Such a result could be explained by the small number of events registered, which lowered the statistical power to detect a possibly significant difference.

The exploratory analysis concerning MRI prediction of locoregional relapse identifies that the most important feature in T2-weighted MR images to predict loco-regional recurrence is signal intensity, with a smaller contribution coming from LAHGLE and total volume.

The best fitting provided an AUC of 0.91 ± 0.18, which may look like an impressive result, but could also be a case of overfitting. More data is needed to perform a validation of the fit and draw conclusions. On the other hand, the interpretation of PCA components can be informative about the most significant radiomic feature. The PCA is built to project (*explain*) the maximum amount of variance in the original data set for each component. Thus, in PCA, the first component explains most of the variance, the second explains most of the remaining variance, and so on. The contribution of each radiomic feature to any PCA component is known, so we can infer which are the relevant features for our classification. In the best fit, the number of PCA components is three, and the first component explains more than 99% of the variance in the dataset. The main contributing features are: Total energy for the first component, contributing to 98% of the component; GLSZM's LAHGLE for the second component (99%); Voxel Volume and Mesh Volume for the third component (49% and 48%). Total Energy is a measure of mean pixel value or signal intensity. The GLSZM is a second-order statistic on the pixel values, and its LAHGLE measures the proportion in the image of the joint distribution of larger size zones with higher gray-level values. Voxel Volume and Mesh Volume are two slightly different measures of GTV total volume. Our interpretation of such results takes into consideration that SI on T2 sequences could be representative of tumor-stroma architecture, tumor permeability, or peri-tumoral inflammation. A relative low signal intensity could be interpreted as a sign of tissue hypercellularity and a more crammed, hypovascular organization (28). GLSZM's LAHGLE could be a manifestation of tumor heterogeneity. As reported by Owczarczyk et al., tumor heterogeneity could be related to more radioresistant behavior (13).

One of the proposed strategies to increase local control is dose-escalated RT, as proposed by the ongoing ACT V seamless pilot trial. The observation of slow-responding biology of the tumor, raises doubt on the usefulness of the RT boost strategy of disease persistence in the last days of RT. Moreover, the impact on local control of doses over 54-56 Gy is unknown, while the possible burden on acute and late toxicity is concrete, including an impairment in sphincteric function (5, 29).

In our view, such a strategy needs to be integrated with novel predictive factors of poor response. MRI predictive tools could help to discriminate poor responders or radioresistant tumor biology. In such a context, an intriguing question is if a radiomic signature predicting poor response could be identified on MRI “*ab initio*” or during or at the end of treatment (30). Given the unique slow-responding tumor biology, pre-treatment MRI has been the point of start of our analysis, and our preliminary results seem to support the presence of a common MRI signature “*ab initio*” in poor responders. More data are clearly needed to find reliable parameters identifying a high-risk patient class, but such patients could be the candidates to be included in the design of a personalized medicine dose escalation clinical trial. In fact, in such a category, the probability of healthy tissue harm from dose-escalation could be balanced with the possibility of benefit in local control. Toxicity of dose escalation could be mitigated also thanks to new RT delivery techniques, such as MRI-LINAC with its unique capacity of online adaptation based on soft tissue high quality onboard imaging (31).

This study has several limitations. Although the results of our statistics seem to appear robust, given the treatment evolution during the years, other factors such as better locoregional staging or better management of side effects could have contributed to the results. Moreover, our predictive model lacks external validation, and we did not manage to include an analysis of functional parameters such as Diffusion Weighted Imaging (DWI).

There are also some strengths in our series. This is a large picture of the pattern of management evolution in a high volume center, able to depict the impact of relatively new techniques on key outcomes in ASCC. Clinical and technical risk factors taken together with radiomic high risk features could open the possibility of personalized medicine for ASCC inside a clinical trial.

## Conclusions

Our series shows that chemotherapy and particularly the evolution in radiotherapy technique have independently improved the prognosis of ASCC patients over the years while reducing acute GI and skin toxicity. IMRT and daily 3D image guidance may be considered standard of care in the management of ASCC. Compliance with CHT and avoidance of RT unplanned interruption seems to be crucial. Therefore, simultaneous supportive care strategies need to be improved in order not to compromise the outcome. Careful comprehensive clinical and radiological evaluation may underline the presence of fistula, given the possible impact on tumor microscopic spread. HIV patients under HAART may be treated with the best available therapeutic regimen given the similar prognosis to HIV negative patients. As local relapse represents the main pattern of failure, a combination of three pre-treatment MRI parameters, such as low signal intensity (SI), a high Gray Level Size Zone Matrix’s Large Area High Grey Level Emphasis (GLSZM’s LAHGLE), and GTV volume, shows a positive correlation with the risk of relapse. Our analysis shows that risk depends on a combination of the three parameters, which in our hypothesis could be integrated in risk stratification to identify candidates for RT dose escalation to be enrolled in clinical trials.

## Data availability statement

The raw data supporting the conclusions of this article will be made available by the authors, without undue reservation.

## Ethics statement

The studies involving human participants were reviewed and approved by ethics committee of Brescia. The patients/participants provided their written informed consent to participate in this study.

## Author contributions

MLB: study conception, design, conduction and writing, contouring planning and delivery of treatments, and statistics. SLM: study conduction and writing. NS: study conduction and writing, contouring. CT: data extraction and exploratory analysis. LS: data extraction and performed exploratory analysis. FT: contouring planning and delivery and follow up. FBa: study conduction, contouring planning, and delivery. PV: planning and delivery of treatments and follow up. FF: study conduction and contouring. AG: planning and delivery of treatments and follow up. LT: study conduction, contouring planning, and delivery. DT: contouring planning and delivery of treatments. VM: contouring planning and delivery of treatments. JI: contouring planning and delivery of treatments and follow up. JA: biopsy, staging, and salvage surgery performer. BF: staging of patients (MRI, Endorectal ultrasound) CT staging, and contouring MRI GTV. FP: CT staging and follow-up. LG: staging of patients (MRI, Endorectal ultrasound) and contouring MRI. NP: biopsy, staging, and salvage surgery performer. LN: study design, conduction, and writing. DA: staging of patients and follow (PET CT). FBe: staging of patients and follow (PET CT). SM: study conception, design, conduction, and writing. MB: study conception, design, conduction, and writing. All authors contributed to the article and approved the submitted version.

## Conflict of interest

The authors declare that the research was conducted in the absence of any commercial or financial relationships that could be construed as a potential conflict of interest.

## Publisher’s note

All claims expressed in this article are solely those of the authors and do not necessarily represent those of their affiliated organizations, or those of the publisher, the editors and the reviewers. Any product that may be evaluated in this article, or claim that may be made by its manufacturer, is not guaranteed or endorsed by the publisher.
